# Characterization of the SNAG and SLUG Domains of Snail2 in the Repression of E-Cadherin and EMT Induction: Modulation by Serine 4 Phosphorylation

**DOI:** 10.1371/journal.pone.0036132

**Published:** 2012-05-02

**Authors:** Patricia Molina-Ortiz, Ana Villarejo, Matthew MacPherson, Vanesa Santos, Amalia Montes, Serhiy Souchelnytskyi, Francisco Portillo, Amparo Cano

**Affiliations:** 1 Departamento de Bioquímica, Facultad de Medicina, Universidad Autonoma de Madrid (UAM), Instituto de Investigaciones Biomédicas “Alberto Sols” CSIC-UAM, IdiPAZ, Madrid, Spain; 2 Karolinska Biomics Center, Department of Oncology-Pathology, Karolinska Institutet, Stockholm, Sweden; Technische Universität München, Germany

## Abstract

Snail1 and Snail2, two highly related members of the Snail superfamily, are direct transcriptional repressors of *E-cadherin* and EMT inducers. Previous comparative gene profiling analyses have revealed important differences in the gene expression pattern regulated by Snail1 and Snail2, indicating functional differences between both factors. The molecular mechanism of Snail1-mediated repression has been elucidated to some extent, but very little is presently known on the repression mediated by Snail2. In the present work, we report on the characterization of Snail2 repression of *E-cadherin* and its regulation by phosphorylation. Both the N-terminal SNAG and the central SLUG domains of Snail2 are required for efficient repression of the *E-cadherin* promoter. The co-repressor NCoR interacts with Snail2 through the SNAG domain, while CtBP1 is recruited through the SLUG domain. Interestingly, the SNAG domain is absolutely required for EMT induction while the SLUG domain plays a negative modulation of Snail2 mediated EMT. Additionally, we identify here novel in vivo phosphorylation sites at serine 4 and serine 88 of Snail2 and demonstrate the functional implication of serine 4 in the regulation of Snail2-mediated repressor activity of *E-cadherin* and in Snail2 induction of EMT.

## Introduction

**Figure 1:**
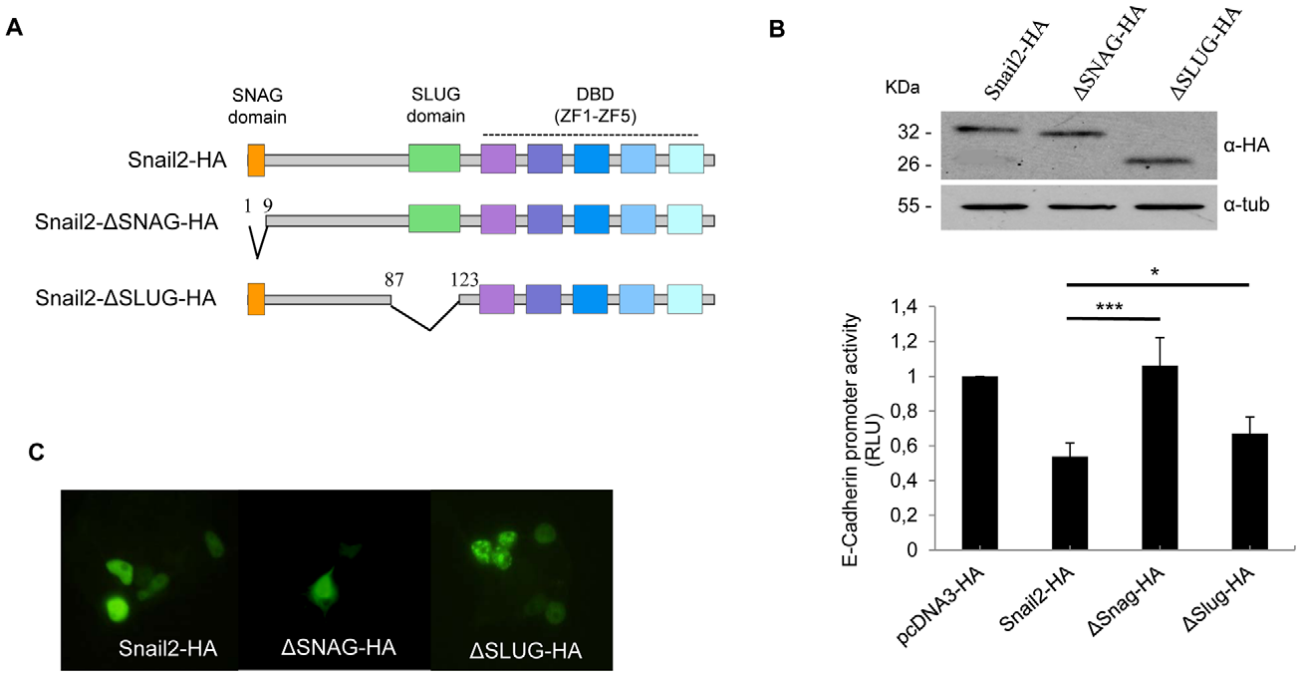
The SNAG and SLUG domains of Snail2 are required for efficient repression of *E-cadherin* promoter. (A) Schematic representation of the organization of mouse Snail2 protein. Upper, the different domains are represented with a color code: the SNAG domain, 1–9 amino acids (orange) the SLUG domain (96 to 123 amino acids) (green) and the five zinc fingers (blue to magenta) constituting the DNA binding domain (DBD). Middle and bottom, schematics of the ΔSNAG and ΔSLUG mutants with indication of the specific amino acids deleted. (B) The repressor activity of Snail2-HA wild type and the indicated mutants on the mouse *E-cadherin* promoter was analyzed on HEK293T cells. Reporter assays were performed with 100 ng of the indicated vectors as described in Material and Methods, and relative luciferase units (RLU) normalized to the activity obtained in the presence of a void control pcDNA3 vector. Results show the mean of triplicate experiments, performed on quadruplicate samples, +/− s.d. *p<0.05; ***p<0.001. Upper insets show western blot controls for equal expression of the different Snail2 forms. (C). Nuclear localization of Snail2-HA and the SNAG and ΔSLUG mutants in transiently transfected HEK293T cells as determined by immunofluorescence analysis.

Snail1 and Snail2 belong to the Snail superfamily of zinc finger transcription factors [1] and have emerged as important repressors of *E-cadherin* and inducers of epithelial to mesenchymal transition (EMT) [2]–[4]. Vertebrate Snail1 and Snail2 factors share a high degree of homology at the DNA binding C-terminal region, containing four and five C2H2 zinc fingers, respectively, and at the N-terminal region that contains the SNAG transactivation domain [5]. The SNAG domain was originally described as a repressor motif present in several vertebrate zinc finger proteins, including Snail and Gfi factors [6], [7]. The minimal SNAG sequence conserved among vertebrate and invertebrate Snail members extends to the first N-terminal 9 amino acids [1]. The SNAG domain is, nevertheless, lacking in Drosophila melanogaster Snail (dSnail), the founder member of the Snail family that instead contains a distinct N-terminal region (NT) and two CtBP (C-terminal binding protein) interacting domains (CID) [8]. There are no CID sites in other Snail members, although two highly degenerate CID sites are present in vertebrate Snail2 [1], [4], [8]. Snail1 and Snail2 present a similar modular organization of nuclear import sequences, distributed among several zinc fingers [9]. However, Snail1 and Snail2 markedly differ in the proline-serine rich central region: Snail1 contains a destruction box and a nuclear export sequence [10], [11], whereas Snail2 contains a specific 28 amino-acid sequence (amino acids 96 to 123), called the SLUG domain (SLUG) of unknown function [1], [5] (see scheme in Fig. 1A).

Previous studies have demonstrated the functional equivalence of Snail1 and Snail2 as EMT inducers and *E-cadherin* repressors, through interaction with proximal E-boxes or the E-pal element in the human or mouse *E-cadherin* promoters, respectively [12]–[16]. They also act on other epithelial genes, such as the tight junction protein claudin1, also through binding to proximal E-boxes on regulatory regions [17]. Nevertheless, significant differences between Snail1 and Snail2 have been observed in their in vitro binding affinity to the E-pal element of the mouse *E-cadherin* promoter [15], and in their ability to repress E-cadherin in distinct breast carcinoma cells and tumours [14], [18], [19]. In addition, gene expression profiling analyses of MDCK cells overexpressing Snail1 or Snail2 demonstrated the regulation of both common and specific genes by both factors [20], indicating relevant biological differences between Snail1 and Snail2. Importantly, functional knockdown studies also revealed a specific role for Snail1 and Snail2 in the tumorigenic and metastatic behaviour of squamous carcinoma cells [21]. The biological differences between both factors are also highlighted by the distinct effect of genetic deletion of Snail1 or Snail2 genes in embryonic development: Snail1 knockout mice are embryonic lethal [22] while Snail2 knockout mice are viable [23]. A differential involvement of Snail1 and Snail2 in neural crest induction and migration during development has also been reported in different species [24]–[26].

The molecular basis for the functional differences between Snail1 and Snail2 factors remains unexplored to date. Thus, while the molecular mechanisms involved in Snail1 mediated repression have been examined in some detail very little is known of the mechanisms operating in Snail2 repression. Several co-repressors and epigenetic remodelling complexes are recruited by Snail1 through the SNAG domain, including mSin3A/histone deacetylase 1/2 (HDAC1/HDAC2) complexes [27], Polycomb repressor complex 2 (PRC2) [28], the Ajuba family of Lim proteins and protein arginine methyl transferase 5 (PRMT5) [29], [30], and the histone lysine specific demethylase 1 (LSD1) [31], [32]. In the case of dSnail1, Ebi1 (the homologue of mammalian TBL1, a component of the NCoR/SMRT-HDAC3 co-repressor complex) is recruited through the distinct NT domain and collaborates with CtBP1 recruited through the conserved CIDs [33], [34]. Regarding Snail2, an initial report demonstrated the requirement for an extended SNAG region (up to 32 N-terminal amino acids) for Snail2 transcriptional repression [8], and more recent studies showed the participation of the SNAG domain in the recruitment of Ajuba proteins by Snail2 [29], [35]. Interestingly, Snail2 has been described to repress several genes through recruitment of CtBP1 and HDAC1 to their proximal promoters in human breast carcinoma cells [36]–[38] but the Snail2 regulatory regions involved in those interactions have yet to be defined. On the other hand, no functional studies on the specific SLUG domain of Snail2 have been reported to date.

Post-translational modifications, in particular phosphorylation events, have been shown to regulate the functional activity of Snail factors, in particular of Snail1. Both negative and positive regulation of Snail1 stability and functional activity by different kinases has been described in different systems [10], [11], [39]–[43]. In contrast, almost nothing is known on post-translational modifications of Snail2, with exception of its interaction and further ubiquitinilation with the F-box protein Ppa [26] or with Mdm2 [44] to control its stability.

In the present report, we have analyzed the functional implication of the SNAG and SLUG domains of Snail2 and investigated the recruitment of different co-repressors. Both domains are required for Snail2 transcriptional repression and for the recruitment of NCoR and CtBP1 co-repressors. In vivo experiments show the absolute requirement of the minimal SNAG domain (1–9 amino acids) for EMT induction and its contribution to Snail2 stability. The influence of Snail2 phosphorylation has also been investigated, leading to the identification of in vivo phosphorylation of serines 4 and 88 and to the characterization of the functional implication of Snail2 serine 4 in modulation of EMT. These data provide new insights into the molecular mechanisms of Snail2 mediated repression and its regulation that might help to explain the observed biological and functional differences of Snail1 and Snail2 factors.

## Results

### Functional characterization of the SNAG and SLUG domains of Snail2

We decided to study the function of SNAG and SLUG domains of Snail2 to further understand the molecular mechanisms of Snail2 repressor activity. For this purpose, we generated deletion mutants of both regions of Snail2 (Figure 1A) and analysed their repression activity on the *E-cadherin* promoter. It is worth mentioning that the SNAG region has been considered in different studies to extend to the first N-terminal 20 amino acids; however, the minimum conserved SNAG region in vertebrate and invertebrate Snail proteins consists only of the first 9 amino acids [1]. Deletion of the minimal SNAG domain fully abolished the Snail2 repression activity on the *E-cadherin* promoter (Figure 1B), in agreement with the function described for the SNAG domain of Snail1 [13], [27], [31]. Surprisingly, deletion of the SLUG domain significantly impaired the repression activity of Snail2 on the *E-cadherin* promoter (Figure 1B). The activity of the different mutants on the *E-cadherin* promoter was similar when analyzed in HEK293T (Human Embryonic Kidney Transformed with adenovirus) cells or in MDCK (Madin Darby Canine Kidney) cells (Figure 1B and Figure S1A, C). Similar results were also obtained for the claudin1 promoter (data not shown). Immunofluorescence analyses of transiently transfected MDCK and HEK293T cells revealed no changes in the subcellular localization of Snail2 when the SNAG or the SLUG domains were deleted (Figure 1C and Figure S1B, C), excluding alterations in nuclear localization as an explanation of the deregulated promoter activity exhibited by both Snail2 deletion mutants. As expected, deletion of both SNAG and SLUG domains, as represented by the ΔNt mutant, leads to full abolition of Snail2 repressive activity on the *E-cadherin* promoter in both HEK293T and MDCK cells (Figure S1C). These results indicate that the minimal SNAG domain is essential for the repressor activity of Snail2 and that the SLUG domain is required for an efficient Snail2-mediated repression.

### NCoR and CtBP1 are functional co-repressors of Snail2

Previous studies have described the interaction of mammalian Snail1 with mSin3A co-repressor through the SNAG domain [27], while in the ancestor dSnail the unique NT region interacts with Ebi co-repressor, the homologue of mammalian TBL1, and cooperates with CtBP recruited through the two conserved CID domains [34]. Therefore, we focus our analysis of Snail2 co-repressors on mSin3A, NCoR and CtBP co-repressors.

**Figure 2:**
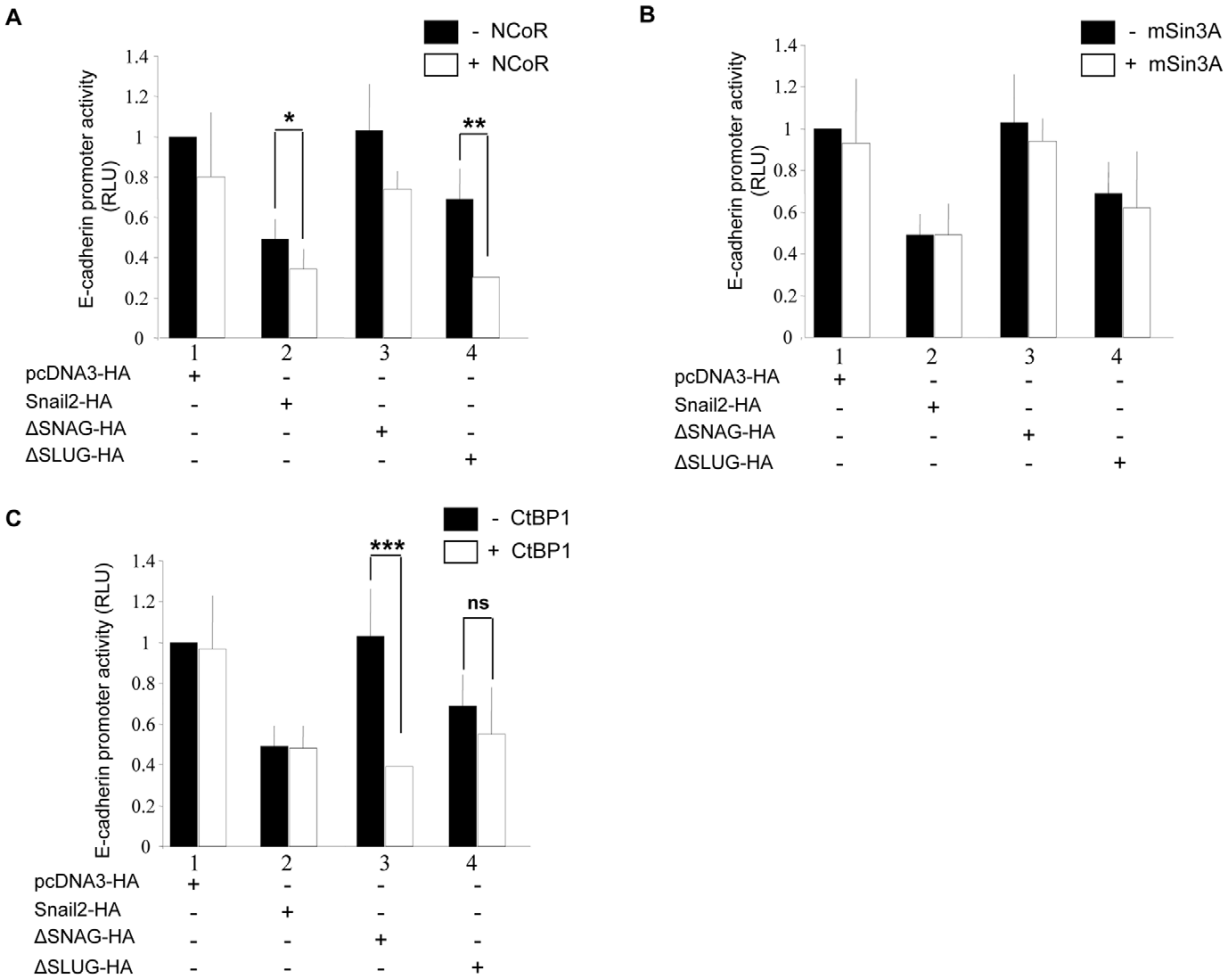
NCoR and CtBP1 act as functional co-repressors of Snail2 depending on the SNAG and the SLUG domains. The co-repressor action of NCoR (A), mSin3A (B) and CtBP1 (C) in the Snail2-mediated repression on the mouse *E-cadherin* promoter was analyzed on HEK293T cells. The *E-cadherin* promoter activity was determined in the presence of 50 ng of Snail2-HA wild type, the ΔSNAG or the ΔSLUG mutants and, when indicated, in the presence of 50 ng of NCoR, mSin3A, or CtBP1. Reporter assays were performed as described in Material and Methods, and relative luciferase units (RLU) normalized to the activity obtained in the presence of a void control pcDNA3-HA vector. Results show the mean of at least triplicate experiments, performed on quadruplicate samples, +/− s.d. *p<0.05; **p<0.005; ***p<0.001; n.s, not significant.

To analyze the functional implication of mSin3A, NCoR or CtBP1 in Snail2 repression, *E-cadherin* promoter assays were carried out in the presence of the various co-repressors. Co-expression of NCoR increased the repression capacity of Snail2 wild type on the *E-cadherin* promoter (Figure 2A, columns #2) while co-expression of mSin3A or CtPB1 had no significant effect on the repression of *E-cadherin* promoter by Snail2 (Figure 2B and C, columns #2). We then investigated the role of the SNAG and SLUG domains in the Snail2 repression activity with different co-repressors. No change in the activity of either mutant was observed in the presence of mSin3A (Fig. 2B, columns 3 and 4). The ΔSNAG mutant was also mainly inactive in the presence of NCoR (Fig. 2A, columns 3) but, surprisingly, exhibits a strongly increased repressor activity in the presence of CtBP1 (Figure 2C, columns #3). On the other hand, the ΔSLUG mutant maintained a moderate repressor activity in the presence or absence of CtBP1 (Figure 2C, columns #4) but exhibited strongly increased repressor activity in the presence of NCoR (Figure 2A, columns #4). Collectively, these data support the requirement of the minimal SNAG domain for NCoR recruitment and/or functional co-repression and suggest a potential negative modulation of the SNAG and SLUG domains in the function and/or interaction of Snail2 with of CtBP1 and NCoR, respectively.

**Figure 3:**
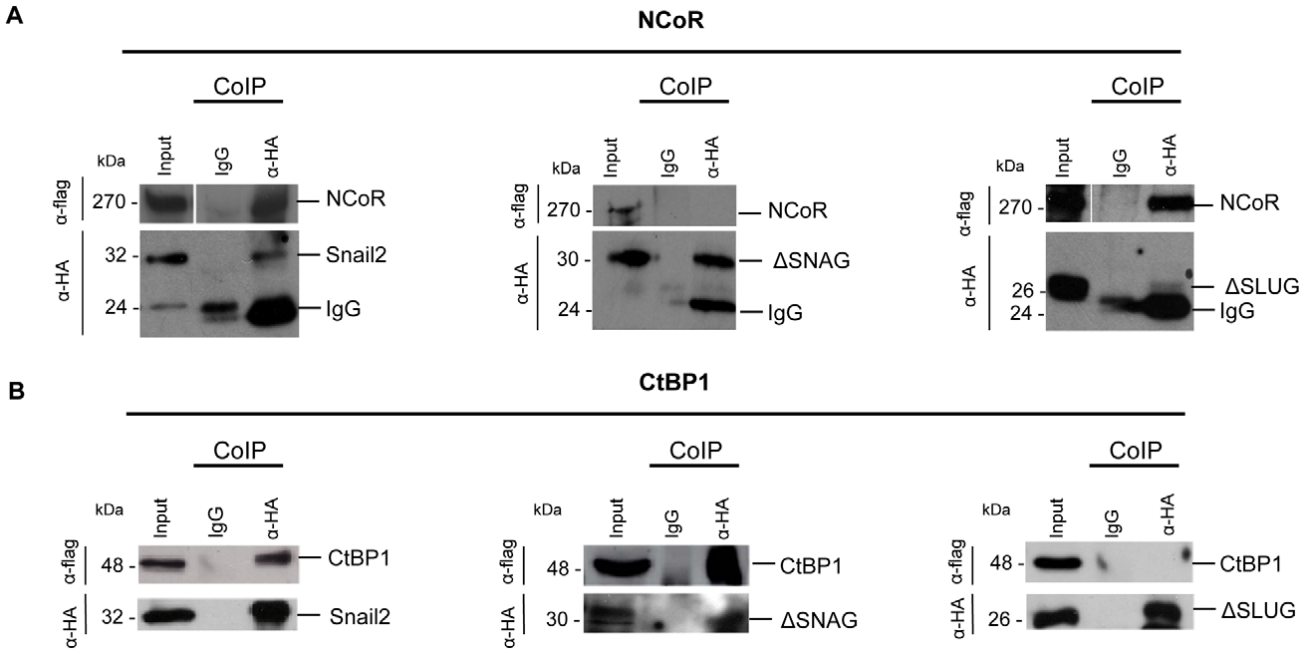
The SNAG and SLUG domains of Snail2 are required for recruitment of NCoR and CtBP1 co-repressors. Co-immunoprecipitation analyses of Snail2-HA wild type (left) and the indicated mutants (middle and right) with (A) NCoR-flag and (B) CtBP1-flag after transient cotransfection in HEK293T cells. Immunoprecipitates were obtained with anti-HA antibodies or control IgG and sepharose G beads (A) or with anti-HA affinity matrix (B), followed by Western blot with anti-flag and anti-HA to detect NCoR and Snail2 (A) or CtBP1 and Snail2 (B). Inputs for transfected NCoR-flag, CtBP1-flag and Snail2-HA (w.t or mutants) are shown in the left lane of each individual panel. Loading controls for IgG Ips are shown in the upper panels; loading control IgG cannot be detected in the case of anti-HA immunoprecipitates (lower panels), because they are retained inside the high affinity anti-HA matrix.

To further understand the function of the SNAG and SLUG domains in the recruitment of co-repressors, Snail2 interaction with NCoR and CtBP1 co-repressors was then analysed by co-immunoprecipitation assays in transiently transfected HEK293T cells. Results indicated that Snail2 interacts with NCoR and CtBP1 (Figure 3A, B, left). Deletion of the SNAG domain completely blocked the Snail2 interaction with NCoR (Figure 3A, middle), but strongly increased the Snail2 interaction with CtBP1 (Figure 3B, middle, compare to Figure 3B, left). On the other hand, deletion of the SLUG domain fully abolished the Snail2 interaction with CtBP1 (Figure 3B, right panel) but it did not affect NCoR interaction (Figure 3A, right). These results indicate that the SNAG domain of Snail2 is required for recruitment of NCoR while the SLUG domain appears to be mainly involved in the recruitment of CtBP1.

### Functional characterization of the SNAG and SLUG domains in Snail2 mediated EMT

**Figure 4:**
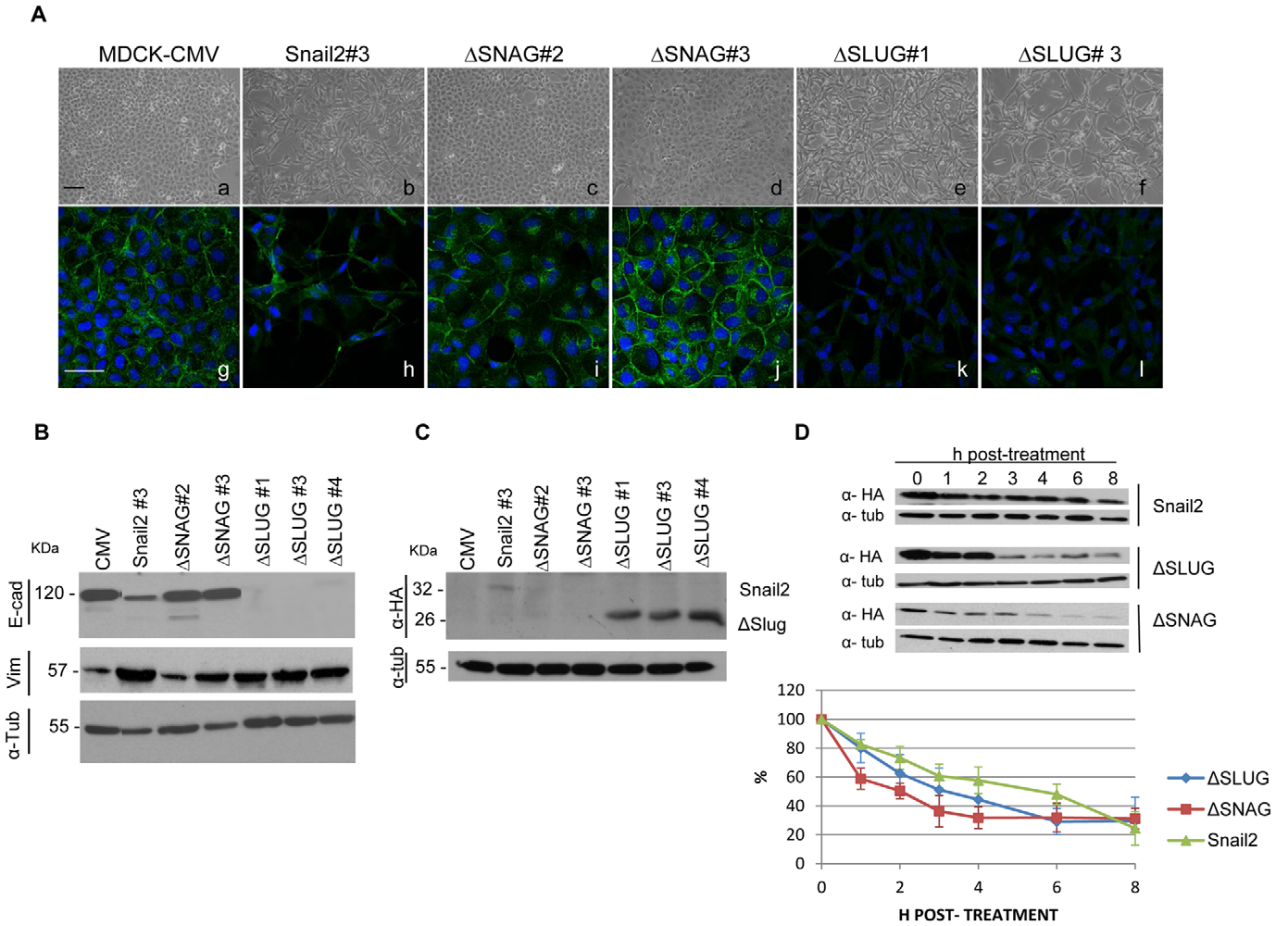
The SNAG domain is absolutely required for Snail2-mediated EMT while the SLUG domain has a negative modulatory action. Snail2-HA wild type and the Snail2-ΔSNAG or the Snail2-ΔSLUG mutants were stably transfected into MDCK cells and selected clones analyzed for phenotype and EMT markers. (A) Phenotypic characterization of one representative Snail2-HA clone (#3), two representative Snail2-ΔSNAG clones (#2 and #3) and two representative Snail2-ΔSLUG clones (#1 and #3) compared to control MDCK-CMV (CMV) cells. Panels a to f, phase contrast images; g to l, immunofluorescence staining for E-cadherin (green). Nuclei are stained with DAPI (blue). Bars, 200 µm. (B) Western blot analysis of the indicated clones for E-cadherin (upper) and vimentin (middle); migration of the indicated proteins is shown in kDa at the left. (C) Western blot analysis of the indicated clones for Snail2-HA, wt or mutants, detection using anti-HA antibodies. Migration of Snail2-HA wild type (32 kDa) and the ΔSLUG mutant (26 kDa) is indicated. α -tubulin (55 kDa) was used as loading control in (B) and (C). Similar results were obtained for all isolated clones. (D) The stability of Snail2-HA and the indicated Snail2-HA mutants in HEK293T cells was determined by incubation in the presence of the translational inhibitor cycloheximide for the indicated time periods. Upper, Western blot analyses of Snail2-HA (wt and mutants) levels of one representative experiment with anti-HA antibodies; α-tubulin was used as a loading control. Bottom, densitometric quantifications of the relative amount of Snail2-HA and the indicated mutants at the indicated time points. Results show the mean of three independent experiments +/− s.d.

To further ascertain the biological relevance of the SNAG and SLUG domains of Snail2 we analysed the availability of the corresponding mutants to induce EMT in vivo. To this end, stable transfectants expressing Snail2-HA wild type and either the ΔSNAG-HA or ΔSLUG-HA mutants were generated in epithelial MDCK cells. We choose MDCK cells for these studies because they represent prototypical epithelial cells in which EMT changes can easily be discerned at both morphological and molecular levels [12], [20], [41], [45], in contrast to HEK293T that exhibit an epithelioid phenotype and low cytoplasmic content making more difficult to distinguish clear phenotypic changes. As previously reported for untagged Snail2 [15], expression of wild type Snail2-HA in MDCK cells induced a phenotypic change compatible with an EMT process as observed from the spindle morphology (Figure 4Ab), decreased E-cadherin expression, increased expression of mesenchymal markers, such as vimentin, and reorganization of the actin cytoskeleton (Figure 4Ah and 4B, and data not shown). Strikingly, stable expression of the ΔSNAG mutant fully abolished the EMT-inducing activity of Snail2, as MDCK-ΔSNAG cells remained fully epithelial both at the phenotypic level and from marker expression (Figure 4Ac,d,i,j, and 4B). In contrast, stable expression of the ΔSLUG mutant induced a full EMT process, even stronger than that induced by Snail2 wild type, as ascertained by the marked spindle and more elongated phenotype of MDCK-ΔSLUG cells (Figure 4Ae,f), which showed complete suppression of E-cadherin and increased vimentin expression (Figure 4Ak,l and 4B). Western blot analysis of the ectopic Snail2-HA proteins in the various transfectants indicated high expression levels of the ΔSLUG mutant and very low or almost undetectable levels of Snail2-HA wild type or the ΔSNAG mutant, respectively (Figure 4C). The low expression level of Snail2-HA wild type in the stable MDCK transfectants is in agreement with previous observations [15]. In general, the level of expression of the various Snail2 mutants is partly coincident with their ability to induce the EMT program. These observations also suggested differences in the protein stability of the various Snail2 mutants. To test this possibility, the stability of the different mutants was analysed by cycloheximide pulse-chase assays. This study showed that, as previously reported in Xenopus [26], Snail2 is a labile protein in human cells with an estimated half-life of about 6 h in HEK293T cells (Figure 4D). Interestingly, the ΔSNAG mutants showed marked decreased protein stability (estimated half-life: 2 h) while the ΔSLUG mutant exhibited and intermediate stability (estimated half-life: 4 h) compared to Snail2 wild type (Figure 4D).

Together, these results indicate that the minimal SNAG domain, apart from being essential for efficient NCoR co-repressor recruitment and transcriptional repressor activity, it is absolutely required for EMT induction and confers stability to Snail2 factor.

### Snail2 is phosphorylated in vivo at serines 4 and 88

**Figure 5:**
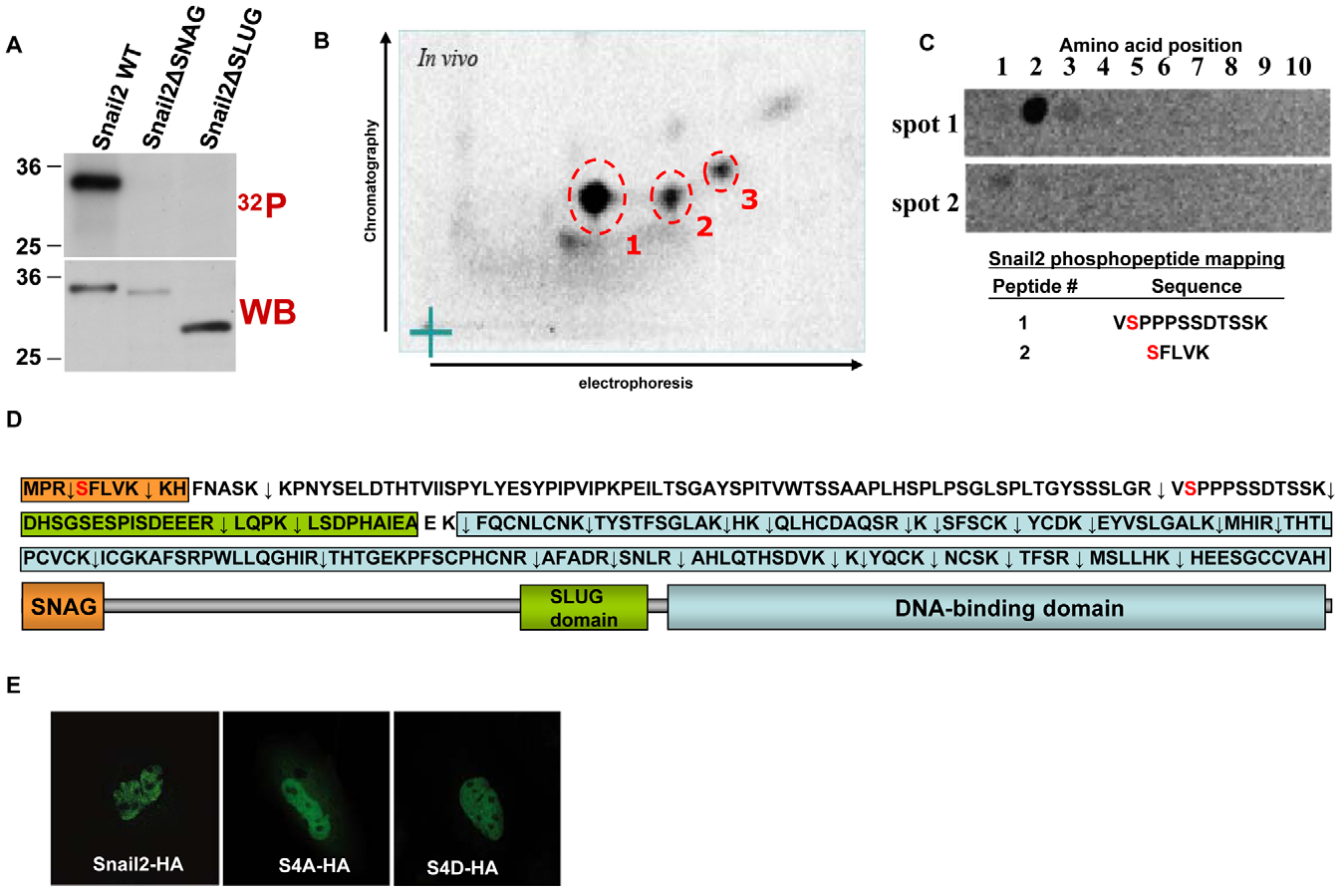
Snail2 is phosphorylated *in vivo* at several potential serine residues. *In vivo* phosphorylation of Snail2-HA was analyzed after transient transfection in HEK293T cells. (A) Autoradiagraphy on immunoprecipitates obtained with anti-HA antibodies from HEK293T cells transfected with Snail2-HA wild type, the ΔSNAG or the ΔSLUG mutants, labeled with 32P-orthophosphate (up); parallel Western blot analysis with anti-HA antibodies (bottom) of lysates from 32P-labelled cells. (B) Two-dimensional phosphopeptide analysis of immunoprecipitated 32P-Snail2-HA after typsin digestion. The main phosphopeptides detected are indicated by circles and numbered 1 to 3. (C) Edmann degradation analyses of the indicated Snail2-32P-phosphopeptides. The amino acid position from the N-terminal of the different peptides is numbered from 1 to 10 above. The sequence of the assigned peptides with the phosphorylated residue in red is indicated below. (D) The amino acid sequence of Snail2 and the tryptic peptides generated, indicated by arrows, is represented; the different domains of Snail2 are indicated by the same color code as in Figure 1A. (E) Nuclear localization of Snail2-HA and the S4A and S4D mutants in transiently transfected MDCK cells as determined by immunofluorescence and confocal analysis.

Post-translational modifications of Snail1 have emerged as key regulatory events that influence Snail1 protein nuclear translocation, stability and/or repression action [3], [46], [47]. However, the potential regulatory role of Snail2's phosphorylation has not been investigated to date. To characterize Snail2 phosphorylation we firstly carried out in vivo phosphorylation studies of Snail2 in transiently transfected HEK293T cells and performed phosphopeptide mapping. Phosphorylated Snail2 appears as a main band (Figure 5A, up) with slightly lower mobility than bulk Snail2 as ascertained from the parallel Western blot control (Figure 5A, bottom). In vivo phosphorylated Snail2 was subjected to trypsin digestion, and the resulting peptides separated in two-dimensions by electrophoresis and chromatography. The resultant phosphopeptide map showed a minimum of three distinct phosphopeptides (Figure 5B). Each phosphopeptide was extracted from the cellulose plate and subjected to Edmann's degradation. This analysis indicated that phosphopeptide 1 was phosphorylated on the 2nd residue and phosphopeptide 2, most likely, phosphorylated at position 1 although the signal was much weaker due to difficulties in extraction and elution (Figure 5C). Phosphopeptide 3 could not be efficiently eluted to allow reliable sequencing.

Comparison of the phosphorylated residues detected in phosphopeptides 1 and 2 with all potential tryptic peptides produced by trypsin digestion of Snail2 (Figure 5D), together with bi-dimensional mobility analysis predictions, strongly suggested that serine 88 and serine 4 were the phosphorylated residues present in posphopeptide 1 and 2, respectively (Figure 5C, D). Interestingly, serine 4 is located inside the repressor SNAG domain while serine 88 is adjacent to the SLUG domain of Snail2. Deletion of either the SNAG or SLUG domains abolished the in vivo phosphorylation potential of Snail2 (Figure 5A), indicating the requirement of both domains for efficient phosphorylation and further supporting the participation of serine 4 and serine 88 in Snail2 phosphorylation.

### Functional characterization of serine 4 in Snail2 mediated EMT

**Figure 6:**
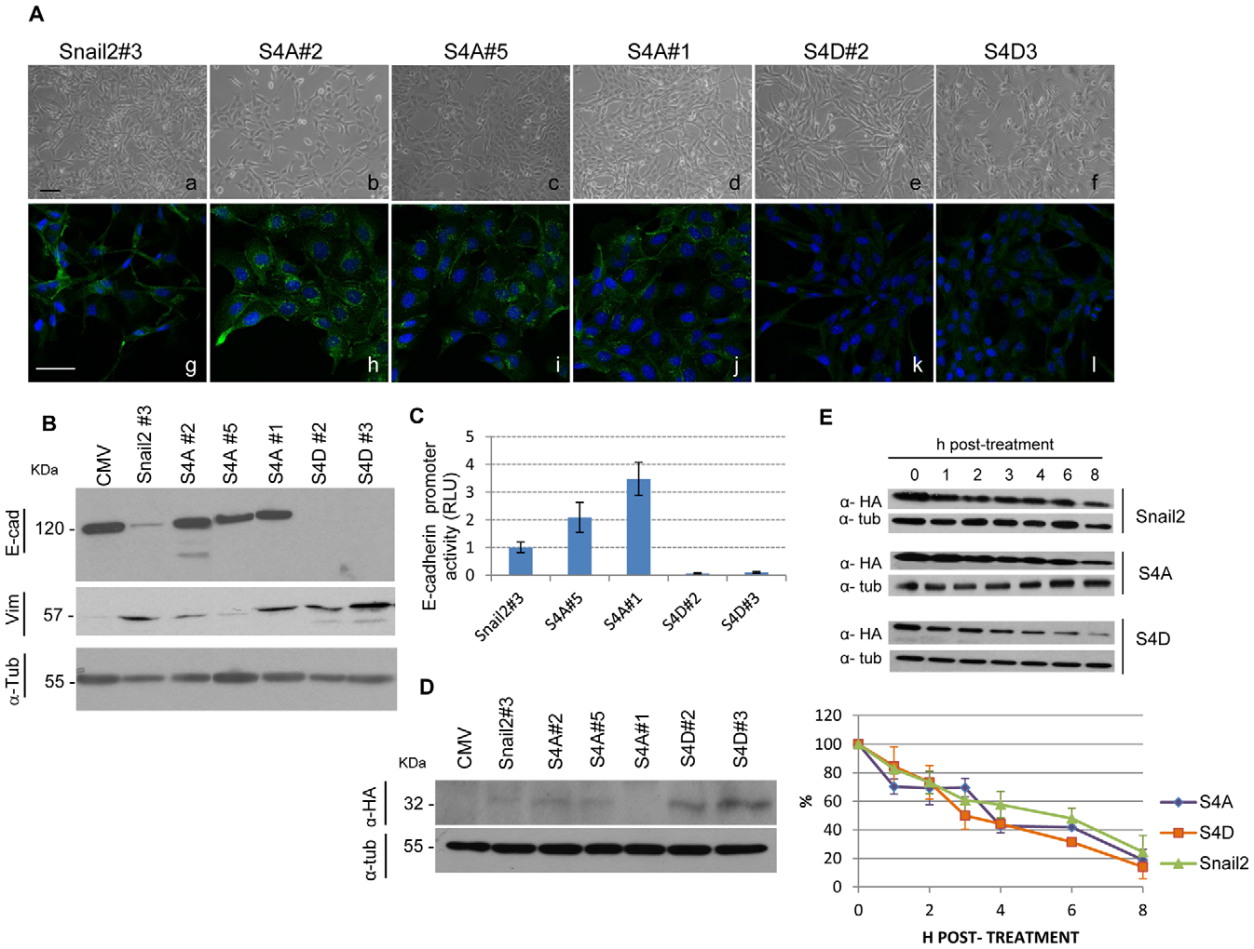
Serine 4 modulates Snail2-mediated EMT. Snail2-HA wild type and the Snail2-S4A or the Snail2-S4D mutants were stably transfected into MDCK cells and selected clones analyzed for phenotype and EMT markers. (A) Phenotypic characterization of three representative Snail2-S4A clones (#1, #2 and #5) and two representative Snail2-S4D clones (#2 and #3) compared to Snail2-HA cells (clone #3). Panels a to f, phase contrast images; g to l, immunofluorescence staining for E-cadherin (green). Nuclei are stained with DAPI (blue). Bars, 200 µm. (B) Western blot analysis of the indicated clones for E-cadherin (upper) and vimentin (middle); migration of the indicated proteins is shown in kDa at the left. (C) *E-cadherin* promoter activity in the indicated MDCK clones. Reporter assays were performed as described in Material and Methods, and relative luciferase units (RLU) normalized to the activity obtained in MDCK-Snail2-HA cells. Results show the mean of duplicate experiments, performed on triplicate samples, +/− s.d. (D) Western blot analysis of the indicated clones for Snail2-HA, wt and mutants, detection using anti-HA antibodies. Migration of Snail2-HA wild type and mutants (32 kDa) is indicated at the left. α-tubulin (55 kDa) was used as loading control in (B) and (D). Similar results were obtained for all isolated clones. (E) The stability of Snail2-HA and the indicated Snail2-HA mutants in HEK293T cells was determined by incubation in the presence of the translational inhibitor cycloheximide for the indicated time periods. Upper, Western blot analysis of Snail2-HA levels of one representative experiment from Snail2-HA wild type and mutants with anti-HA antibodies; α-tubulin was used as a loading control. Bottom, densitometric quantification of the relative amounts of Snail2-HA and the two mutants at the indicated time points. Results show the mean of three independent experiments +/− s.d.

Because the previous observations supported the relevance of the SNAG domain for Snail2 function, we focused our attention on the potential involvement of serine 4 in Snail2 mediated EMT. Non-phosphorylatable (S4A) and phosphomimetic (S4D) mutants of Snail2 were obtained and analysed for their EMT induction capacity in MDCK cells as compared to Snail2 wild type. Initially, we confirmed that both Snail2-S4A and Snail2-S4D mutants are localized to the cell nuclei when transiently transfected in MDCK cells (Figure 5E). Interestingly, stable expression of the Snail2-S4A mutant induced a partial EMT process by which MDCK-S4A cells acquired a spindle-like morphology at low confluence, similar to that induced by Snail2 wild type (Figure 6Ab–d, compare to Figure 6Aa) but maintained high levels of E-cadherin expression (Figure 6B), partly organized at cell-cell contacts at high cell density (Figure 6Ah–j), and low to moderate levels of vimentin expression (Figure 6B). Surprisingly, stable expression of the S4D mutant induced a remarkable EMT phenotype, manifested by strong spindle morphology (Figure 6Ae,f), complete absence of E-cadherin and increased vimentin expression (Figure 6Ak,l and 6B). Results of *E-cadherin* promoter activity in the different transfectants were fully consistent with the observed partial/full EMT phenotype and E-cadherin levels, showing complete promoter repression in Snail2-S4D, even stronger than in Snail2 wild type cells, and moderate activity in Snail2-S4A expressing cells (Figure 6C). Analyses of the protein levels of the ectopic Snail2 proteins in the stable transfectants showed very low levels of the Snail2 wild type and Snail2-S4A proteins and higher levels of Snail2-S4D protein in the corresponding transfectants (Figure 6D). Cycloheximide pulse-chase assays of the Snail2-S4A and Snail2-S4D mutants showed that both proteins have similar stability (estimated half-life: 4 h) slightly lower than Snail2 wild type (estimated half-life: 6 h) (Figure 6E). Those data discard that differences in protein stability between Snail2 wild type and the S4 mutants may account for the different protein levels detected in the corresponding stable transfectants. Instead, they could suggest that Snail2-S4D is best tolerated by MDCK cells than Snail2 wild type or S4A mutant, and can provide a cell survival advantage compared to Snail2 wild type or the Snail2-S4A mutant. Taken together, the results obtained with the Snail2-serine 4 mutants indicate that integrity of serine 4 is required for Snail2 to induce an efficient EMT process that can be further influenced by the serine 4 phosphorylation status.

## Discussion

Snail factors have emerged as essential regulators of physiological and pathological EMT processes [1], [2], [4], [46], [47]. Studies in embryogenesis and cell systems have indicated similar but non-overlapping roles for Snail1 and Snail2 factors in EMT induction during gastrulation and neural crest formation [16], [22]–[25] as well as in target gene regulation [20]. Furthermore, a distinct role for both Snail factors has been described in carcinoma cells and tumours, including colorectal and gynaecological carcinomas and melanomas [3], [4], as well as in the tumorigenic and metastatic behaviour of squamous carcinoma cells [21]. The molecular bases underlying the biological differences between Snail1 and Snail2 have so far remained elusive. While several co-repressors and chromatin remodelling components are recruited by Snail1 [27]–[31], almost no information exists on the co-repressor(s) collaborating in Snail2-mediated repression.

Using the *E-cadherin* promoter as a prototypic model for Snail factor repression we have examined here the function of the SNAG and SLUG domains in Snail2-mediated repression and co-repressors recruitment. Our studies demonstrated that the minimal SNAG domain is essential for Snail2-mediated repression, as in Snail1 repression [13], [27], and is absolutely required for EMT induction. On the other hand, the SLUG domain is required for efficient Snail2-mediated repression. Using interaction and promoter activity assays, we have characterized the interaction of Snail2 and its functional cooperation with NCoR and CtBP1 co-repressors. NCoR is recruited to Snail2 through the SNAG domain while recruitment of CtBP1 depends on an intact SLUG domain (Figure 3). The SLUG domain also appears to participate in the regulation of recruitment and/or functional activity of the SNAG-recruited co-repressors since its deletion significantly increases the NCoR co-repressor activity (Figure 2A). Notably, the Snail2/CtBP1 interaction and functional collaboration seem to be hindered by the SNAG domain since active co-repression by CtBP1 can only be observed following deletion of the SNAG region (Figure 2C). This suggests that the SLUG domain may present a cryptic conformation in the native Snail2 protein and that conformational changes as those likely induced by SNAG deletion or potential modifications could favour interaction with CtBP1 or other co-repressors. The present data are in agreement with previous reports indicating the participation of CtBP1 in *E-cadherin* repression [48] and the description of Snail2/CtBP1/HDAC1 complexes in Snail2 repression of several target genes, such as BRCA2, VDR and UbcH5c, in breast cancer cells [36]–[38], and provide additional information on the SLUG domain. Whether CtBP1 interacts directly with the SLUG domain of Snail2 or if such interaction is mediated through intermediary factors, as previously suggested [49], remains to be established. Further, the present study indicates that NCoR acts as a functional co-repressor of Snail2 while mSin3A does not (Figure 2), in contrast to its observed role in Snail1-mediated repression [27]. These observations provide a rationale for the differences observed between Snail1 and Snail2 in *E-cadherin* repression in different contexts. We speculate that differential expression of distinct co-repressors (NCoR, mSin3A and/or CtBP1) may influence the function and determine the apparent prevalent role of Snail1 or Snail2 under specific contexts, as previously suggested [50]. Future studies in other cellular models and tumours will be required to validate this hypothesis.

The present results also demonstrate that the minimal SNAG domain plays a positive role in Snail2 stability, as previously reported for the expanded SNAG domain (1–20 amino acids) of Snail1 [31], further delimiting the SNAG region to the first 9 amino acids as required to confer stability, co-repressor recruitment and EMT induction to Snail proteins. Whether the SNAG region controls stability by influencing the Snail2 interaction with Ppa, or other components of the proteasome machinery, remains to be established, although notably the Ppa interacting region of Xenopus Slug or that of the ortolog FBXL14 with human Snail1 has been located outside the SNAG domain [26], [51]. Our results also support a negative modulation of the SLUG region in Snail2 function, since the Snail2-ΔSLUG expressing cells exhibit a stronger *E-cadherin* repression and EMT phenotype than the Snail2-wild type expressing cells (Figure 4). It is thus tempting to speculate that the SLUG domain of Snail2 confers a conformational state to Snail2 that hinders the interaction of the SNAG domain with is cognate co-repressors to promote active repression and effective EMT induction. This agrees with the reported lower repression potency of Snail2 compared to Snail1 [15].

We may speculate that differential contextual signals influence the recruitment of active co-repressor complexes to Snail1 and Snai2 factors, thus influencing the function of both factors in distinct biological contexts. Post-translational modifications of mammalian Snail1 have been shown to modulate Snail1 stability and functional repressor activity. In particular, phosphorylation by GSK3β or PKD1 plays a negative role [11], [40], [42] while phosphorylation by PAK1, CK2, PKC or Lats2, or interaction with lysyl oxidase-like 2/3 (LOXL2/3) exert a positive effect on Snai1 functionality [39], [41], [43], [52]. In contrast, almost no information exists on post-translational modifications of Snail2 with the exception of its interaction with and modulation of stability by Mdm2 [44] and the F-box protein Ppa [26], recently reported to regulate also the stability of other EMT inducers in *Xenopus* [53]. We now describe that phosphorylation of Snail2 within the SNAG domain at serine 4 plays a regulatory action on Snail2 mediated induction of EMT. The strong EMT program induced by stable expression of the phosphomimetic Snail2-S4D mutant and the partial EMT induced by stable expression of the Snail2-S4A mutant suggests that integrity of serine 4 of Snail2, and thus its potential phosphorylation, is required for induction of a full EMT program by Snail2 in in vivo contexts, in agreement with the strict requirement of the SNAG domain for Snail2-mediated *E-cadherin* repression and EMT induction.

**Figure 7:**
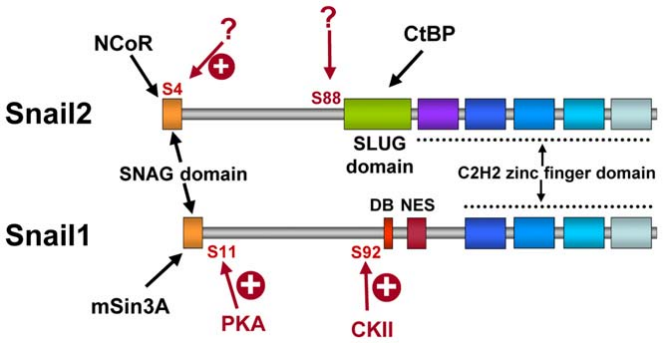
Snail1 and Snail2 recruit distinct co-repressors and may be modulated by distinct phosphorylation events. Schematic diagrams showing the structural domains of Snail2 (up) and Snail1 (bottom), indicating that the SNAG domain is involved in the recruitment of NCoR and mSin3A co-repressors in Snail2 and Snail1, respectively, while the Slug domain recruits CtBP1 in the case of Snail2. The regulatory phosphorylated serine residues identified in Snail2 in the present study and in Snail1 in a previous one [41] are also shown with indication of the kinases involved in the case of Snail1.

Since serine 4 is conserved in the SNAG domain of all vertebrate Snail factors [1], [5], it is highly likely that phosphorylation of serine 4 plays a similar regulatory role on Snail1 as well, although further studies are required to formally support this point. In this context it is worth considering the potential differences in the regulatory phosphorylation events in Snail1 and Snail2. We recently described activating phosphorylation events at serine 11 and serine 92 in Snail1 [41]. Interestingly, serine 11 is not conserved in Snail2 while the conserved serine 92 does not appear phosphorylated in Snail2 but instead presents in vivo phosphorylation at serine 88 supporting the existence of differential regulatory mechanisms between Snail1 and Snail2 (Figure 7). Although we could not detect a functional role for serine 14 or serine 88 of Snail2 (data not shown), further studies are required to define more precisely the potential regulatory differences between Snail1 and Snail2 by phosphorylation at the SNAG region and other regulatory regions, as the recently described activating phosphorylation at threonine 203 in Snail1 [43].

In summary, the present data support the existence of distinct molecular mechanisms for Snail1 and Snail2 mediated repression on *E-cadherin* and identify new regulatory phosphorylation events in Snail2 of biological relevance for EMT. This work also demonstrates the importance of the SNAG and SLUG domains in the repression and functional activity of Snail2. These findings shed light on the molecular basis underlying reported differences in repression and biological behaviour between Snail1 and Snail2 factors that can contribute to specific therapies targeting each factor.

## Materials and Methods

### Generation of plasmids and expression vectors

The pcDNA3-Snail2-HA plasmid containing mouse Snail2 cDNA was generated by subcloning of the previously described pcDNA3-mSlug [15] and has been used as a template for site-directed mutagenesis and generation of deletion constructs. Site directed mutagenesis on the indicated serine residues was performed as described [54]. The ΔSNAG (deletion of 1–9 amino acids) and ΔNt (deletion of 1–125 amino acids) mutants were generated by PCR amplification of the region of interest from the pcDNA3-Snail2-HA vector, whereas the ΔSLUG (deletion of 87–123 amino acids) mutant was generated by digestion of two intermediate vectors obtained by point mutation in each of the selected nucleotides to be deleted that also introduced an internal EcoRV site. EcoRV/Xho digested fragments from each vector were purified and ligated to obtain the vector with the deleted sequence. The specific oligonucleotide sequences used for mutagenesis and generation of deletion vectors are provided in Table S1. After mutagenesis and subcloning, the entire Snail2-HA cDNA was sequenced to verify that only the nucleotide changes introduced by the mutagenic oligonucleotides or the specific deleted regions were obtained. The pSC2-mSin3A-myc expression vector was previously described [27]; the pcDNA3-flag-CtBP1 expression vector (coding for mouse CtBP1) was kindly provided by Dr. K. Verschueren (Flanders Interuniversitary Institute of Biotechnology, Belgium) and pcDNA3-NCoR-flag (coding for mouse NCoR) by Dr. A. Aranda (Instituto de Investigaciones Biomédicas “Alberto Sols” CSIC-UAM, Madrid, Spain).

### Cell culture and transfections

Established HEK293T and MDCK cells were obtained from the ATCC collection (LGC Standards-SLU, Barcelona, Spain) according to the following catalogue references: CRL-11268TM (HEK293T) and CRL-2936TM (MDCK.2). As previously described [12], [27], both cell lines were maintained in DMEM medium supplemented with 10% foetal calf serum and antibiotics (100 µg/ml ampicillin, 32 µg/ml gentamicin, Sigma-Aldrich, St Louis, MO). Stable and transient transfections were performed using lipofectamine reagent (Invitrogen, Carlsbad, CA) according to manufacturer's instructions. For generation of stable clones, MDCK cells (1×106 cells) grown in P60 plates were transfected with the indicated Snail2-HA wild type, mutant constructs, or control pcDNA3-HA construct (CMV) and grown in the presence of G418 (500 µg/ml) for two to three weeks; individual colonies were then selected and grown individually. At least ten independent clones were selected and characterized from each transfection with the pcDNA3-Snail12-HA mutants; two independent clones were selected from pcDNA3-Snail2-HA wild type or control transfections.

### Co-immunoprecipitation and Western blot assays

Co-immunoprecipitation analyses were performed as described previously [27], [41]. Basically, HEK293T cells were transiently transfected with the indicated vectors for 48 h. Lysates were then obtained in immunoprecipitation buffer (50 mM Tris-HCl, pH 8.0, 150 mM NaCl, 5 mM EDTA, 0.5% NP-40) containing protease and phosphatase inhibitors (2 µg/ml aprotinin, 1 µg/ml leupeptin, 1 mM PMSF, 1 mM sodium vanadate, 10 mM sodium fluoride) and precleared with Sepharose G-beads. Supernatants were subjected to overnight incubation with anti-HA affinity matrix (Roche, Indianapolis, IN) or Sepharose G-beads coated with anti-rat IgG as an immunoprecipitation control. Immunoprecipitates were resolved in 7.5–12% PAGE-SDS gels, transferred to membranes, and incubated with the indicated antibodies. Membranes were finally developed using ECL reagent following manufacturer's instructions (Amersham, Picataway, NJ). Blots were incubated with rat anti-HA (Roche; 1∶100) or mouse anti-flag (Sigma; 1∶3,000). The secondary antibodies used were HRP-coupled goat anti-rat (Pierce, Rockford, IL, 1∶10,000) or sheep anti-mouse (Pierce, 1∶1,000). For detection of E-cadherin, vimentin and Snail2-HA expression, Western blots were performed on whole-cell lysates obtained as previously described [45] using rat anti-E-cadherin ECCD2 mAb (1∶200, produced in our laboratory from the ECCD2 hybridoma, a gift of M. Takeichi, Ricken Center, Japan), mouse anti-vimentin (1∶500, Dako) or rat-anti HA (Roche), followed by HRP-coupled secondary antibodies.

### Protein stability assays

Analysis of Snail2-HA wild type and the indicated mutant proteins was performed in the presence of cycloheximide as previously described [41]. Basically, HEK293T cells were transiently transfected with the indicated vectors and 24 h later cells were treated with 20 mM cycloheximide (Sigma-Aldrich) for the indicated time intervals. Cells were lysed in RIPA buffer (0.1% SDS, 0.5% sodium deoxycholate, 1% NP-40, 150 mM NaCl, 50 mM Tris-HCl, pH 8.0), containing protease and phosphatase inhibitors and the expression of Snail2-HA analyzed by Western blotting as described above. Three independent experiments were performed; the mean +/− s.d. of the integrated and normalized band intensity is represented in the graphs.

### Immunofluorescence

Cells grown on coverslips were rinsed with PBS, fixed with cold (−20°C) methanol 3 min, or 3.7% formaldehyde for 20 min at room temperature and permeabilized using 0.5% (v/v) Triton X-100. Transiently or stably transfected MDCK or HEK293T cells were processed for indirect immunofluorescence with rat anti-HA (1∶50) and rat anti-E-cadherin (1∶100) antibodies. Alexa-488 or Alexa-555 (1∶800, Molecular Probes, Eugene, OR) were used as secondary fluorescent antibodies. Nuclei were detected by DAPI stain. Samples were analysed using a confocal SP2 Spectral Leica microscope and a Nikon 90i microscope equipped with epifluorescence and ×63 objective.

### Promoter activity assays

Promoter activity assays were basically performed as previously reported [15], [45]. The mouse *E-cadherin* promoter construct (−178 to +92), upstream of firefly luciferase, was transiently transfected in 24-well plates (200 ng/well) together with pCMV-βGal (Promega, Madison, WI) (10 ng/well) for transfection efficiency control. When indicated, co-transfections were carried out in the presence of the indicated amount of pcDNA3-Snail2-HA wild type, pcDNA3-Snail2-HA mutants and/or the indicated amounts of co-repressors (mSin3A, NCoR and/or CtBP1). Luciferase and βGal activities were measured using the Dual-luciferase β-Glo Reporter assay kit (Promega) and normalized to the promoter activity detected in mock transfected (pcDNA3-HA) cells as described [27], [45]. *E-cadherin* promoter activity in stable MDCK-Snail2 transfectants was also analyzed as previously described [15]. Reporter assays in the various experimental settings were performed at least twice and using triplicate samples. The mean +/− s.d is represented.

### *In vivo* phosphorylation

*In vivo* phosphorylation assays were performed as recently described [41]. HEK293T cells were grown in 30-mm-diameter plates and transfected with pcDNA3-Snail2-HA or the indicated Snail2-HA mutants. After 24–36 h, cells were washed three times with DMEM phosphate-free medium, supplemented with 10% foetal calf serum, and subsequently incubated in the same medium containing 1 mCi/ml [32P]orthophosphate (Amersham) for 4 h. Cells were lysed with ice-cold RIPA buffer containing protease and phosphatase inhibitors, the resulting lysate centrifuged at 14,000× g for 15 min, and the supernatant incubated overnight at 4°C with 1 µg anti-HA and 25 µl of protein G-Sepharose (Amersham). Immunoprecipitates were collected and washed four times with ice-cold RIPA buffer. Precipitated proteins were resolved by SDS-PAGE and transferred to nitrocellulose membranes before exposure to x-ray film overnight to view radioactively labelled proteins.

### Phosphopeptide mapping and Edmann's degradation

Phosphopeptide mapping was performed essentially as described [41]. Briefly, phosphorylated Snail2 bands were excised from the membrane and blocked for 30 min in 0.5% polyvinylpirrolidon K30 (Sigma-Aldrich), 0.6% acetic acid at 37°C. After several washes with distilled water, bands were each incubated overnight in 50 mM NH4HCO3, pH 8.0, with 1 µg modified sequencing grade trypsin (Promega, Madison, WI) at 37°C. The solution containing eluted peptides was transferred to a fresh tube and the radioactivity measured to ensure efficient elution. The peptides were frozen and dried by vacuum evaporation and oxidized in performic acid for 1 h on ice in the dark. The oxidization was stopped through dilution in water, the solution was frozen, and vacuum evaporated. The peptides were then washed twice in water, finally resuspended in 10 µl electrophoresis running buffer (2.5% formic acid, 7.5% acetic acid, pH 1.9) and carefully spotted onto a 20× 20-cm cellulose TLC plate (layer thickness 0.1 mm; cat. no. 5716, Merck, Rahway, NJ). Electrophoresis and chromatography was performed following previously described conditions [41]. The results were observed using a Fuji FLA-3000 PhosphorImager (Fuji) and the images adjusted using Adobe Photoshop version 7.0 (San Jose, CA).

For Edmann's degradation, phosphopeptides were eluted from the plates in pH 1.9 electrophoresis buffer and lyophilized. The fractions were then subjected to automated Edmann degradation, using an Applied Biosystems Gas Phase Sequencer model 470A (Foster City, CA) as described [41]. Released phenylthiohydantoin amino acid derivatives from each cycle were spotted onto TLC plates. The radioactivity in each spot was quantitated by exposure to a screen and scanning in a FLA-3000 PhosphorImager (Fuji).

## Supporting Information

Figure S1 **The SNAG and SLUG domains of Snail2 are required for efficient repression of** ***E-cadherin*** **promoter in MDCK cells.** (A) The repressor activity of Snail2-HA wild type and the indicated mutants on the mouse *E-cadherin* promoter was analyzed on MDCK cells. Reporter assays were performed with 100 ng of the indicated vectors as described in Material and Methods, and relative luciferase units (RLU) normalized to the activity obtained in the presence of a void control pcDNA3 vector. Results show the mean of triplicate experiments, performed on quadruplicate samples, +/− s.d. ***p<0.001. (B) Nuclear localization of Snail2-HA and the ΔSNAG and ΔSLUG mutants in transiently transfected MDCK cells as determined by confocal immunofluorescence analysis. (C) Left, Schematic representation of mouse Snail2 wild type and the indicated deletion mutants: ΔSNAG ΔSLUG and ΔNt. Right columns, repression activity on *E-cadherin* promoter (indicated as maximum and minimum percentages in independent experiments) and nuclear localization of the different Snail2-HA proteins in MDCK and HEK293T cells. N.t., not tested.
(TIF)

Table S1 **Sequences of oligonucleotide used for the generation of Snail2-HA mutants.** Sequences of oligonucletides, forward (Fw) and reversre (Rw), for generation of the indicated Snail2-HA mutants by PCR are indicated in pairs. For generation of the ΔSLUG mutant, two intermediate vectors were generated, as indicated in Material and Methods section, containing Ev restriction sites at the indicated positions, corresponding at 87 and 123 amino acids, respectively; the pair of (Fw and Rw) oligonucleotide sequences used for amplification on each corresponding fragment, Ev87 and Ev123 are indicated.
(TIF)
