# Supplementary figures and images for: Characterization of the SNAG and SLUG Domains of Snail2 in the Repression of E-Cadherin and EMT Induction: Modulation by Serine 4 Phosphorylation
Source: PLoS One. 2012 May 2;7(5):e36132. doi: 10.1371/journal.pone.0036132 (PMC3342263; doi:10.1371/journal.pone.0036132)

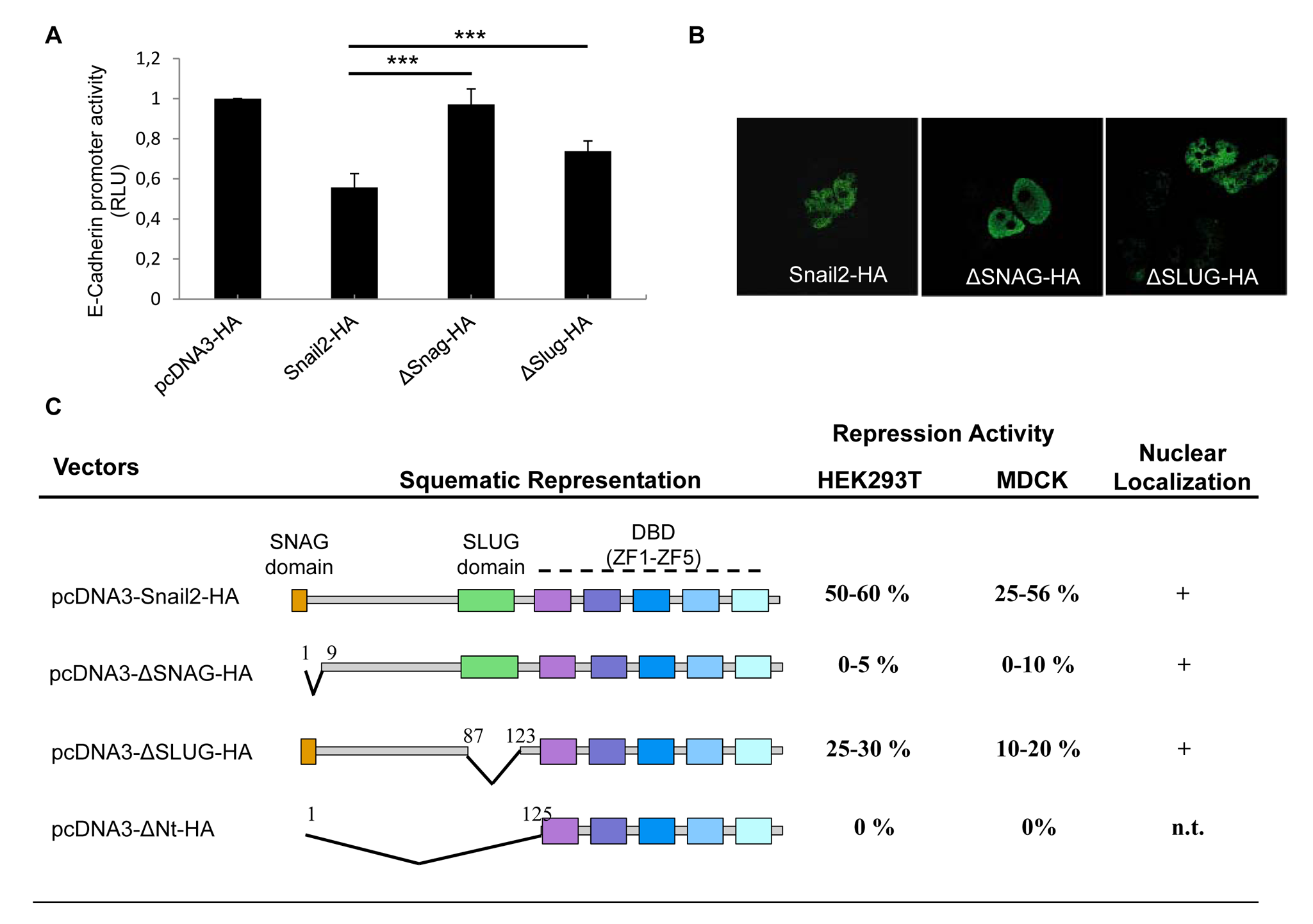

Supplement: Figure S1 — The SNAG and SLUG domains of Snail2 are required for efficient repression of E-cadherin promoter in MDCK cells. (A) The repressor activity of Snail2-HA wild type and the indicated mutants on the mouse E-cadherin promoter was analyzed on MDCK cells. Reporter assays were performed with 100 ng of the indicated vectors as described in Material and Methods, and relative luciferase units (RLU) normalized to the activity obtained in the presence of a void control pcDNA3 vector. Results show the mean of triplicate experiments, performed on quadruplicate samples, +/− s.d. ***p<0.001. (B) Nuclear localization of Snail2-HA and the ΔSNAG and ΔSLUG mutants in transiently transfected MDCK cells as determined by confocal immunofluorescence analysis. (C) Left, Schematic representation of mouse Snail2 wild type and the indicated deletion mutants: ΔSNAG ΔSLUG and ΔNt. Right columns, repression activity on E-cadherin promoter (indicated as maximum and minimum percentages in independent experiments) and nuclear localization of the different Snail2-HA proteins in MDCK and HEK293T cells. N.t., not tested. (TIF) [file pone.0036132.s001.tif]

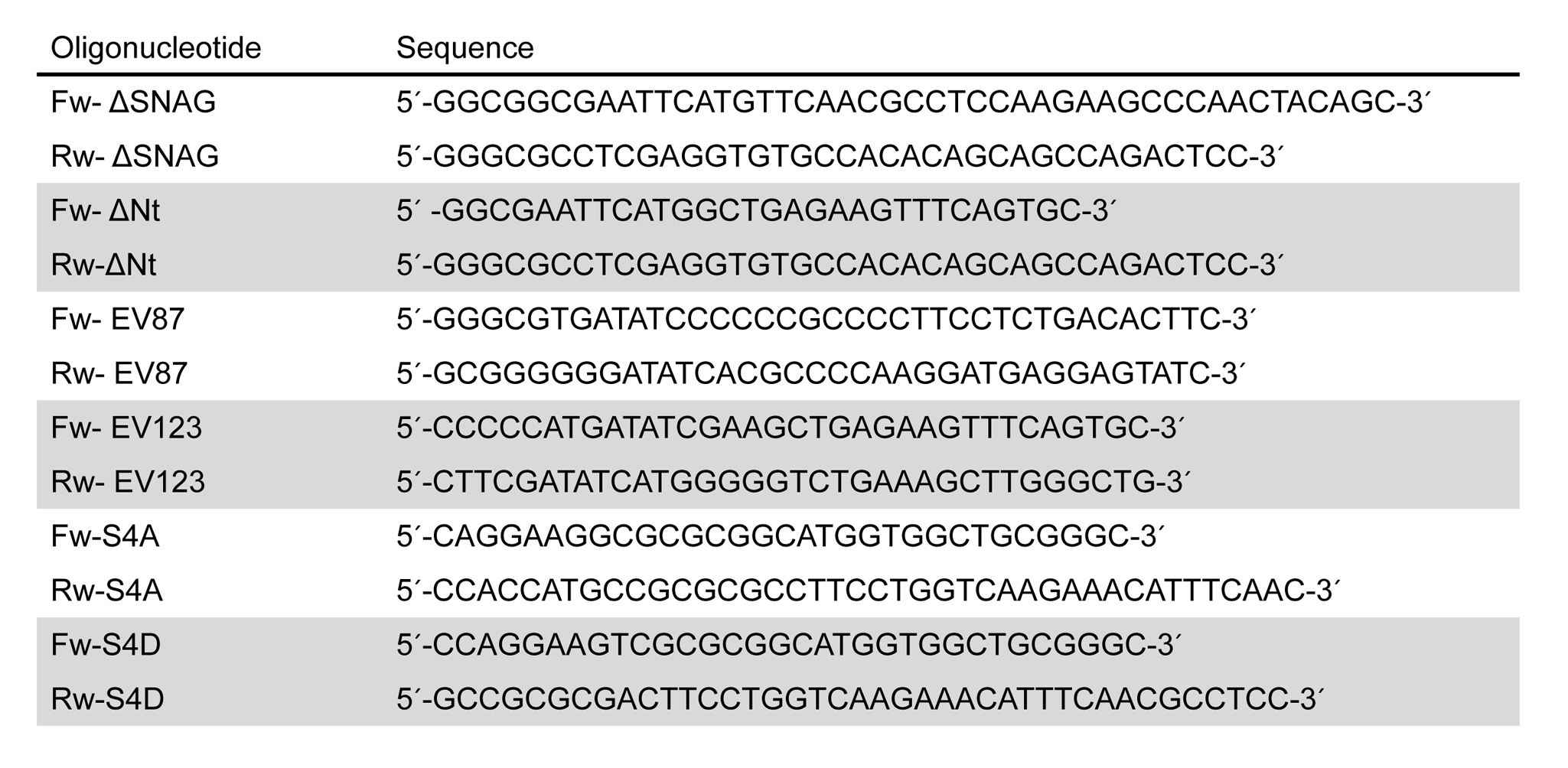

Supplement: Table S1 — Sequences of oligonucleotide used for the generation of Snail2-HA mutants. Sequences of oligonucletides, forward (Fw) and reversre (Rw), for generation of the indicated Snail2-HA mutants by PCR are indicated in pairs. For generation of the ΔSLUG mutant, two intermediate vectors were generated, as indicated in Material and Methods section, containing Ev restriction sites at the indicated positions, corresponding at 87 and 123 amino acids, respectively; the pair of (Fw and Rw) oligonucleotide sequences used for amplification on each corresponding fragment, Ev87 and Ev123 are indicated. (TIF) [file pone.0036132.s002.tif]
